# Contrasting ecological responses of abundant and rare bacterial and fungal subcommunities along an alpine marsh wetland degradation gradient

**DOI:** 10.3389/fmicb.2026.1970129

**Published:** 2026-09-29

**Authors:** Shijia Zhou, Mingzhu Cao, Jiao Tang, Chen Chen, Ni Zhang, Tao Wu, Kelong Chen

**Affiliations:** 1 Qinghai Province Key Laboratory of Physical Geography and Environmental Process, College of Geographical Science, Qinghai Normal University, Xining, China; 2 Key Laboratory of Tibetan Plateau Land Surface Processes and Ecological Conservation (Ministry of Education), Qinghai Normal University, Xining, China; 3 Qinghai Province Key Laboratory of Environmental Geology, Qinghai Provincial Environmental Geological Exploration Bureau, Xining, China

**Keywords:** abundant and rare taxa, alpine marsh wetland, microbial subcommunities, soil metagenomics, wetland degradation

## Abstract

**Introduction:**

Abundant and rare microbial taxa may respond differently to wetland degradation, yet their relative contributions to community reorganization remain poorly resolved in alpine marsh wetlands.

**Methods:**

We investigated lightly (LD), moderately (MD), and severely degraded (SD) alpine marsh wetlands on the northern shore of Qinghai Lake using 15 soil metagenomes and nine soil physicochemical properties. Bacterial and fungal genera were classified as abundant, intermediate, rare, or low-prevalence taxa according to domain-specific prevalence and mean relative abundance. Sample-level richness and original abundance contributions, alpha and beta diversity, community composition, distribution breadth, candidate indicator genera, and environmental associations were compared across the degradation gradient.

**Results:**

Total detected bacterial richness declined from 346.2 genera per sample in LD to 287.0 in SD. The rare bacterial share of detected richness decreased from 45.3% to 40.4%, while its original abundance contribution decreased from 6.79% to 3.70%. Abundant fungi accounted for 97.1–98.5% of total fungal abundance. Rare bacterial richness declined monotonically with degradation, whereas Shannon diversity, Simpson diversity, and Pielou's evenness declined in abundant fungi. All four focal subcommunities differed significantly in composition among degradation levels, with the largest PERMANOVA effect observed for abundant fungi and the smallest for rare fungi. Rare fractions exhibited greater incidence-based beta diversity and narrower observed distributions than abundant fractions. Soil PC1 and PC2 were associated with 51.6%, 41.4%, and 55.1% of the variation in abundant bacterial, rare bacterial, and abundant fungal communities, respectively, but with only 0.6% of the variation in rare fungal communities.

**Discussion:**

Partitioning microbial communities into abundant and rare fractions revealed pronounced abundance- and domain-dependent reorganization along the wetland degradation gradient. Rare bacteria showed marked losses in richness and abundance contribution, whereas abundant fungi exhibited stronger declines in within-community diversity and greater environmental structuring. These contrasting responses were obscured in whole-community summaries, highlighting the value of explicitly separating abundant and rare taxa when evaluating microbial responses to alpine marsh wetland degradation.

## 1 Introduction

Alpine marsh wetlands are critical components of high-altitude ecosystems on the Qinghai–Tibet Plateau and provide essential ecosystem services, including water regulation, carbon sequestration, nutrient cycling, and biodiversity conservation. Owing to their low temperature, high soil moisture, and slow organic matter decomposition rates, alpine wetlands accumulate substantial soil organic carbon and represent important carbon reservoirs in mountain regions (Bai et al., 2010; Yang et al., 2022). However, alpine marsh wetlands across the Qinghai–Tibet Plateau are highly vulnerable to climate change and anthropogenic disturbances, and many of these wetlands have undergone substantial degradation.Alpine marsh wetland degradation is commonly accompanied by vegetation deterioration, hydrological alteration, declining soil moisture, nutrient imbalance, and changes in soil physicochemical conditions, which can modify belowground habitats and ecosystem processes (Zhang et al., 2011; Li et al., 2019). Because soil microorganisms regulate organic matter decomposition, nutrient transformation, and carbon cycling, degradation-induced changes in vegetation and soil environments strongly influence microbial community structure by altering resource availability and environmental filtering (Fierer, 2017; Delgado-Baquerizo et al., 2018). Therefore, elucidating how soil microbial communities respond to alpine marsh wetland degradation is essential for understanding the ecological consequences of environmental deterioration and predicting the stability of high-altitude wetland ecosystems.

Soil microbial communities are characterized by highly uneven abundance distributions, with a relatively small fraction of abundant taxa coexisting with a large number of low-abundance taxa that constitute the microbial rare biosphere (Pedrós-Alió, 2012). Although species classified as abundant taxa generally represent only a limited proportion of total microbial richness, they often account for a major fraction of community biomass and metabolic activity because their high abundance and frequent occurrence increase their contribution to ecosystem processes (Pedrós-Alió, 2006; Lynch and Neufeld, 2015). These taxa are generally considered important contributors to fundamental ecological functions, including organic matter transformation, nutrient cycling, and microbial interactions within local environments (Delgado-Baquerizo et al., 2018). In contrast, rare taxa comprise a substantial proportion of microbial taxonomic diversity and represent a reservoir of phylogenetic and ecological variation that contributes to community-level responses under environmental fluctuations (Shade et al., 2014; Jousset et al., 2017). However, the ecological roles of rare taxa are not solely determined by their low abundance. Rare microorganisms often exhibit narrower ecological niches and stronger habitat specialization than abundant taxa, which may make them particularly sensitive to environmental changes and disturbance gradients (Mo et al., 2018). Conversely, some rare taxa can persist under unfavorable conditions through dormancy, dispersal, and functional redundancy, allowing them to maintain population viability and rapidly increase in abundance when environmental conditions become suitable (Shade et al., 2014; Lynch and Neufeld, 2015). Therefore, abundant and rare subcommunities represent contrasting ecological strategies. Abundant taxa are characterized by high local abundance, frequent occurrence, and broader environmental distributions, which favor persistence across heterogeneous conditions and sustained contributions to ecosystem processes. In contrast, rare taxa generally occur at low abundance and show more restricted distributions and stronger habitat specialization; some can persist through dormancy or spatial refugia and increase rapidly when environmental conditions become favorable, thereby providing a reservoir of microbial diversity and ecological response capacity.

Although microbial responses to ecosystem degradation have been extensively investigated, most studies have focused on total Microbial community, particularly bacterial or fungal communities, there are few studies on the responses of taxa with different abundances to alpine grassland degradation. Previous studies in degraded grasslands, wetlands, and other terrestrial ecosystems have mainly evaluated overall changes in microbial diversity, community composition, and environmental associations, providing important insights into microbial responses to habitat deterioration (Fierer et al., 2007; Delgado-Baquerizo et al., 2018). However, total community analyses may obscure the contrasting ecological behaviors of abundant and rare subcommunities, which may differ in community turnover, niche breadth, and sensitivity to environmental variation (Lynch and Neufeld, 2015). This distinction is important because abundant and rare taxa differ markedly in their distribution patterns, ecological responses, and contributions to community reorganization under environmental change. For example, degradation-gradient studies on the Qinghai–Tibet Plateau have primarily characterized changes in overall bacterial and fungal diversity, community composition, and their associations with vegetation and soil properties (Dong et al., 2021; Xu et al., 2024). This distinction may also be taxon dependent: abundant fungi can occupy broader environmental gradients than rare fungi, indicating potentially different niche strategies between abundance classes (Jiao and Lu, 2020). Studies that simultaneously partition bacterial and fungal communities further indicate that abundant and rare fractions can differ in diversity, assembly processes, and responses to disturbance (Zheng et al., 2021; Wang Z. et al., 2023). However, diversity, community turnover, niche breadth, degradation-stage specificity, and environmental regulation have rarely been integrated within a single framework for alpine marsh wetlands. This gap is particularly relevant because wetland degradation simultaneously alters vegetation structure, soil moisture, nutrient availability, and other physicochemical conditions, potentially imposing different environmental filters on abundant and rare taxa (Xu et al., 2024). Moreover, although abundant–rare microbial ecology has been investigated in Tibetan Plateau grassland soils (Ji et al., 2020), metagenomic evidence explicitly comparing abundant and rare bacterial and fungal subcommunities along alpine marsh wetland degradation gradients remains limited. Resolving this gap is therefore important for determining whether degradation-associated microbial reorganization is expressed predominantly through changes in abundant taxa, rare taxa, or both.

Here, we used a space-for-time LD–MD–SD gradient in the Qinghai Lake Basin and combined 15 soil metagenomes with soil physicochemical measurements. We asked whether the sample-level richness and original abundance contributions of abundant, rare, intermediate, and low-prevalence taxa changed across the gradient; whether alpha diversity and composition responded differently among abundant and rare bacterial and fungal fractions; whether incidence-based beta-diversity components, observed distribution breadth, and degradation-associated genera differed among fractions; and how much compositional variation was associated uniquely or jointly with the measured soil gradient and degradation category. We expected abundant fractions to contribute most of the original abundance and show broader distributions and stronger associations with broad soil gradients, whereas rare fractions would contribute more to richness and show greater sample-to-sample turnover. These hypotheses describe expected tendencies rather than fixed properties of all abundant or rare taxa.

## 2 Materials and methods

### 2.1 Overview of the study area

The study area is located in Gangcha County, Haibei Tibetan Autonomous Prefecture, Qinghai Province, China (Figure 1). Autonomous Prefecture, Qinghai Province, on the northern shore of Qinghai Lake, northeastern Qinghai–Tibet Plateau. The study area extends from 37°16′08″ to 37°16′22″ N and from 100°11′35″ to 100°11′49″ E, at an elevation of approximately 3,000 m above sea level. The region has a plateau continental climate characterized by a large diurnal temperature range, a mean annual temperature of approximately −0.6 °C, a mean July maximum of 11 °C, a mean January minimum of −17.5 °C, and annual precipitation of approximately 370–420 mm. The dominant soil type is chestnut soil. The vegetation is mainly composed of *Carex moorcroftii, Argentina anserina*, and *Elymus nutans*, while salt-tolerant species such as *Suaeda glauca* occur in more severely degraded areas.Vegetation surveys were conducted using five 1 m × 1 m quadrats within each degradation level. Within each quadrat, plant species identity, species cover, and plant height were recorded. Total vegetation cover was visually estimated as the proportion of the quadrat surface occupied by the vertical projection of living vegetation, whereas exposed-ground cover was estimated as the proportion of the quadrat surface not covered by vegetation. Species richness was calculated as the number of plant species recorded within each quadrat.

**Figure 1:**
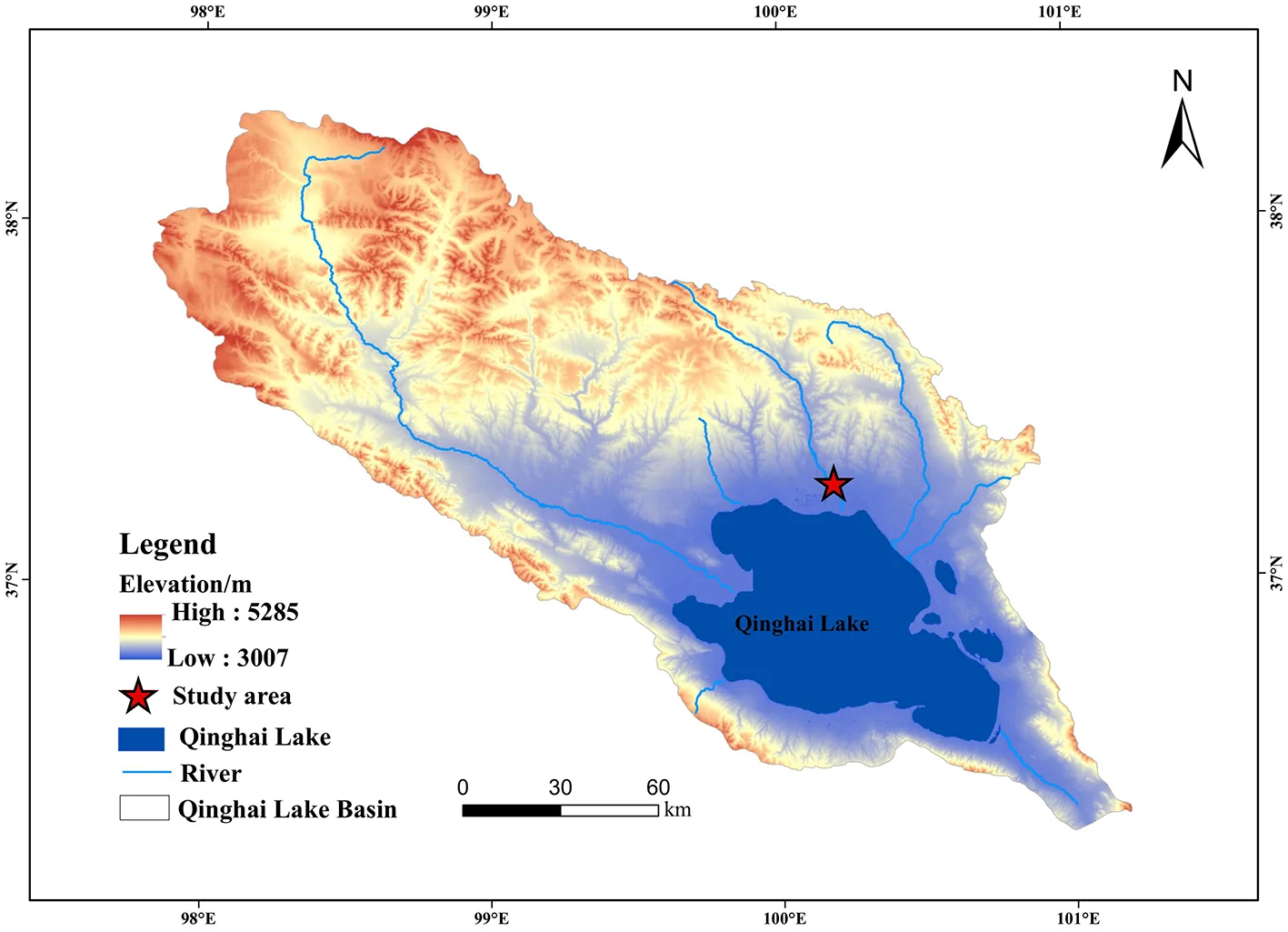
Overview of the study area.

A spatial degradation gradient comprising lightly degraded (LD), moderately degraded (MD), and severely degraded (SD) wetlands was established using vegetation structure, exposed-ground proportion, and plant-community composition as the primary field criteria. LD wetlands were characterized by nearly continuous vegetation cover (98–99%), approximately 1–2% exposed ground, and vegetation dominated by *C. moorcroftii* and *A. anserina*. MD wetlands had intermediate vegetation cover (60–80%), approximately 20–40% exposed ground, and a more heterogeneous graminoid–forb community dominated mainly by *E. nutans* and *A. anserina*. SD wetlands had sparse vegetation cover (15–50%), approximately 50–85% exposed ground, markedly reduced plant height, and scattered communities containing *E. nutans* and the salt-tolerant species *S. glauca*. Soil physicochemical properties were not used to define the degradation categories but were subsequently used to characterize and independently evaluate the environmental gradient.

The three spatial locations were sampled concurrently in June 2025, constituting a space-for-time comparison rather than longitudinal monitoring. Five independent soil plots were established within each degradation category, with at least 2 m between adjacent plots. Within each plot, three soil cores were collected randomly from 0–20 cm and combined into one composite sample. Visible roots, stones, and plant residues were removed. The 15 composite samples were transported on dry ice and stored at −80 °C until DNA extraction.

### 2.2 Soil physicochemical measurements and metagenomic profiling

Nine soil physicochemical properties were included in the environmental analysis: pH, available nitrogen (AN), available phosphorus (AP), available potassium (AK), soil organic carbon (SOC), total carbon (TC), inorganic carbon (IC), soil moisture, and soil temperature. Soil pH was determined potentiometrically in a 1:2.5 soil-to-water suspension. AN was measured using the alkaline hydrolysis diffusion method, AP using NaHCO3 extraction followed by molybdenum–antimony colorimetry, and AK using neutral ammonium acetate extraction followed by flame photometry. SOC was determined using the potassium dichromate–sulfuric acid external-heating oxidation method, and TC was measured using an elemental analyzer. IC was calculated as the difference between TC and SOC. Soil moisture and temperature were recorded during field sampling. Total soil DNA was extracted using the FastDNA SPIN Kit for Soil (MP Biomedicals, USA). DNA integrity was evaluated by agarose gel electrophoresis, and DNA concentration and purity were assessed using a NanoDrop 2000 spectrophotometer. Qualified DNA was fragmented to approximately 400 bp, and sequencing libraries were constructed using the NEBNext Ultra DNA Library Prep Kit for Illumina. Paired-end sequencing was performed on the DNBSEQ-T7 platform using a 2 × 150 bp strategy.

Raw reads were assessed using FastQC v0.11.9 and seqtk v1.3-r106 and filtered using fastp v0.23.2. Reads containing more than one ambiguous base, reads in which fewer than 60% of bases had Phred scores of at least 20, and reads shorter than 100 bp after trimming were removed. Clean reads from each sample were assembled independently using MEGAHIT v1.2.9. Contigs of at least 500 bp were retained for downstream analyses. Protein-coding genes were predicted from the retained contigs using Prodigal v2.6.3, and genes of at least 99 bp were retained. The predicted genes were subsequently pooled and dereplicated using MMseqs2 v15.6f452 to construct the non-redundant gene catalog. Clean reads were mapped to the non-redundant gene catalog using CoverM v0.7.0. Gene abundance was expressed as transcripts per million (TPM), accounting for gene length and library size. Predicted proteins were aligned against the bacterial GTDB and fungal NR reference databases using DIAMOND v2.0.14, and contig-level taxonomic assignments were obtained using CAT_pack v6.0.1. Taxonomic abundance was calculated by summing the TPM values of non-redundant genes assigned to each taxon and then aggregating the results at the genus level. Bacterial and fungal profiles were processed and normalized separately. Within each domain, genus-level relative abundance was calculated as the proportion of the total genus-level TPM in each sample.

### 2.3 Definition of abundant and rare subcommunities

Abundant and rare subcommunities were defined independently for bacteria and fungi using genus-level relative-abundance profiles. A genus-level taxonomic feature was considered detected in a sample when its relative abundance was at least 0.01%. Prevalence was defined as the proportion of the 15 samples in which the feature was detected. Only features occurring in at least three samples, corresponding to a prevalence of at least 20%, were retained for subcommunity classification. Among prevalence-qualified features, abundant taxa were defined as those with a grand mean relative abundance of at least 0.10% across the 15 samples, whereas rare taxa had a grand mean relative abundance below 0.05%. The complete classification of genus-level taxa into abundant and rare subcommunities is provided in Supplementary Table S1. Features with mean relative abundances between 0.05% and 0.10% were classified as intermediate and excluded from direct abundant–rare comparisons. Features with a prevalence below 20% were also excluded. After classification, abundance values below the 0.01% detection threshold were set to zero, and abundant and rare matrices were separately renormalized to sum to one within each sample. These thresholds were selected to separate dominant and low-abundance taxa while minimizing overlap between abundance classes, and their robustness was evaluated using alternative classification criteria. The robustness of abundance-based classification was evaluated using alternative abundance thresholds (Supplementary Table S4).

All genus-level taxonomic features were retained in the abundance-contribution, diversity, community-composition, β-diversity, niche-breadth, and environmental analyses. However, the indicator-genus analysis was restricted to classified genera by excluding labels containing “unclassified”, “uncultured”, “unknown”, “norank”, “unassigned”, or “metagenome”.The robustness of the main classification was examined using alternative abundance and detection thresholds. Sensitivity scenarios included a stricter rare threshold of less than 0.01%, a broader rare threshold of less than 0.10%, a stricter abundant threshold of at least 0.50%, and per-sample detection thresholds of any nonzero abundance and at least 0.001%. For each scenario, the number and abundance contribution of retained taxa, the degradation-related trend in Shannon diversity, and the PERMANOVA effect size were recalculated. Sensitivity-analysis PERMANOVA tests used 999 permutations.

### 2.4 Subcommunity diversity, composition, turnover, and indicator taxa

Observed richness, Shannon diversity, Simpson index, and Pielou's evenness were calculated separately for abundant bacteria, rare bacteria, abundant fungi, and rare fungi. Observed richness was defined as the number of taxa remaining in a sample after application of the 0.01% detection threshold. Shannon diversity was calculated using natural logarithms, the Simpson index was calculated as one minus the sum of squared proportional abundances, and Pielou's evenness was calculated as Shannon diversity divided by the logarithm of observed richness. Because the analyses were based on TPM-derived relative-abundance profiles rather than integer read counts, conventional count-based rarefaction was not applied. The ordered degradation gradient was coded as LD = 1, MD = 2, and SD = 3. Monotonic changes in alpha diversity were evaluated using Spearman's rank correlations, whereas overall differences among the three degradation levels were assessed using Kruskal–Wallis tests. Epsilon-squared was calculated as the effect size for Kruskal–Wallis comparisons. Trend and Kruskal–Wallis probabilities were adjusted separately across the predefined family of 16 alpha-diversity tests. Community composition was evaluated using Bray–Curtis dissimilarities calculated from the separately renormalized subcommunity matrices. Principal coordinate analysis (PCoA) was used to visualize compositional variation. Overall differences among LD, MD, and SD were evaluated using PERMANOVA with 9,999 permutations, with PERMANOVA R^2^ reported as the multivariate effect size. Homogeneity of multivariate dispersion was examined using PERMDISP with 9,999 permutations. Pairwise PERMANOVA tests were subsequently conducted for LD vs. MD, LD vs. SD, and MD vs. SD.

Presence–absence matrices were used to characterize within-group β-diversity. For each pair of samples, total Jaccard dissimilarity was calculated from the numbers of shared and unique taxa. The turnover component was quantified using the Simpson replacement fraction, and the remaining component was treated as nestedness-related dissimilarity. Mean total dissimilarity, turnover, and nestedness were summarized separately within LD, MD, and SD. Because pairwise distances are not statistically independent, these components were interpreted descriptively. Levins' niche breadth was calculated for each taxon from its proportional abundance distribution across the 15 samples. Original taxon abundances, rather than the separately renormalized subcommunity matrices, were used in this calculation after values below the detection threshold were set to zero. Abundant and rare niche breadths were compared separately for bacteria and fungi using two-sided Mann–Whitney U tests, equivalent to Wilcoxon rank-sum tests. Rank-biserial correlations were reported as effect sizes, and the two domain-level probabilities were adjusted using the Benjamini–Hochberg procedure. Indicator values were calculated separately for abundant bacteria, rare bacteria, abundant fungi, and rare fungi. For each classified genus, the indicator value combined its specificity to a degradation level with its frequency of occurrence within that level. The degradation level producing the largest indicator value was assigned as the corresponding indicator group. Significance was evaluated using 9,999 permutations of the LD, MD, and SD labels. Probabilities were adjusted separately within each domain–subcommunity combination, and only genera with an FDR-adjusted *q* value below 0.05 were considered significant indicators. All tested genera were reported in the Supplementary Tables, although only the six strongest indicators for each domain–subcommunity–degradation combination were displayed in the main figure.

### 2.5 Environmental associations and statistical analysis

The nine soil physicochemical variables were standardized to Z-scores before multivariate analysis. Principal component analysis was conducted on the standardized soil matrix, and the first two soil principal components were used to summarize the major environmental gradients while reducing collinearity among the original variables. Distance-based redundancy analysis was subsequently conducted for each of the four subcommunities using the corresponding Bray–Curtis distance matrix as the response and soil PC1 and PC2 as predictors. Model performance was summarized using the unadjusted and adjusted R^2^ values, and significance was evaluated using a pseudo-F test with 9,999 permutations. The four dbRDA probabilities were corrected together using the Benjamini–Hochberg procedure. Because the sample size was limited to 15, individual soil variables were not simultaneously entered into the dbRDA models. Downstream statistical analyses and visualizations were performed in Python using pandas, NumPy, SciPy, and Matplotlib. All permutation analyses used a fixed random seed to ensure reproducibility. For overall PERMANOVA and PERMDISP, probabilities were adjusted separately across the four subcommunity matrices. Pairwise PERMANOVA probabilities were adjusted across the three contrasts within each subcommunity. Indicator-value probabilities were adjusted within each domain–subcommunity combination, and dbRDA probabilities were adjusted across the four models. Statistical significance was defined as an FDR-adjusted *q* value below 0.05.

## 3 Results

### 3.1 Composition and abundance contributions of abundant and rare microbial taxa

Across all abundance classes, mean detected bacterial richness declined from 346.2 genera per sample in LD to 297.6 in MD and 287.0 in SD, whereas fungal richness declined from 53.0 to 50.2 and 47.8 (Figure 2; Supplementary Table S1). In bacteria, rare genera represented 45.3% of detected richness and 6.79% of original abundance in LD, compared with 40.4% and 3.70% in SD. Over the same gradient, the abundant bacterial contribution increased from 84.81% to 91.07%, while its richness contribution increased from 31.8% to 38.9%. Low-prevalence bacterial genera declined from 5.3% to 1.7% of detected richness. In fungi, abundant genera represented 68.5–72.4% of detected richness and 97.06–98.46% of original abundance, whereas rare genera contributed approximately 8% of detected richness and less than 0.7% of original abundance. These original, pre-renormalization summaries show that degradation-related losses were concentrated in low-abundance bacterial richness, while community abundance remained dominated by abundant genera.

**Figure 2:**
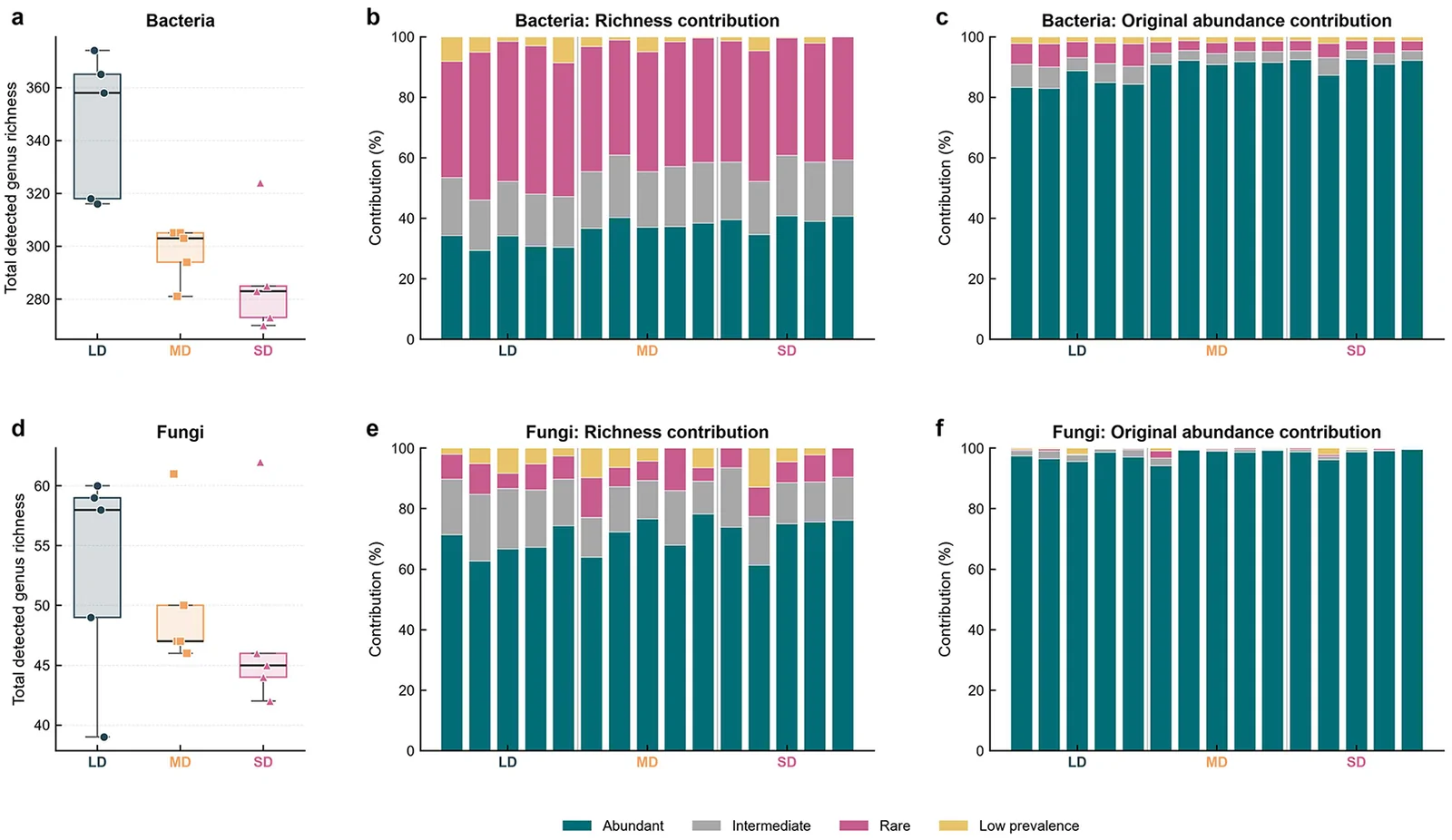
Occupancy–abundance patterns and contributions of abundant and rare microbial taxa. **(a, b)** Prevalence and mean relative abundance of bacterial and fungal genera. Dashed lines indicate the prevalence threshold (20%) and abundance thresholds separating abundant (≥0.10%), intermediate (0.05–0.10%), and rare (<0.05%) taxa. **(c, d)** Numbers of genera and their contributions to total bacterial and fungal abundance. **(e)** Richness contributions of abundant, intermediate, rare, and low-prevalence fungal genera across LD, MD, and SD. **(f)** Original abundance contributions of the four fungal abundance classes across LD, MD, and SD.

### 3.2 Alpha-diversity responses of abundant and rare subcommunities

Alpha-diversity responses differed markedly among subcommunities along the degradation gradient (Figure 3; Supplementary Table S2). Abundant bacterial richness differed among degradation levels (Kruskal–Wallis *q* = 0.037) without a monotonic trend; Dunn tests identified lower richness in LD than MD, whereas SD was intermediate. Rare bacterial richness declined monotonically from 157.6 genera per sample in LD to 116.2 in SD (trend *q* = 0.028), although the three-group Kruskal–Wallis result was not significant after correction. Other bacterial alpha-diversity metrics remained nonsignificant.

**Figure 3:**
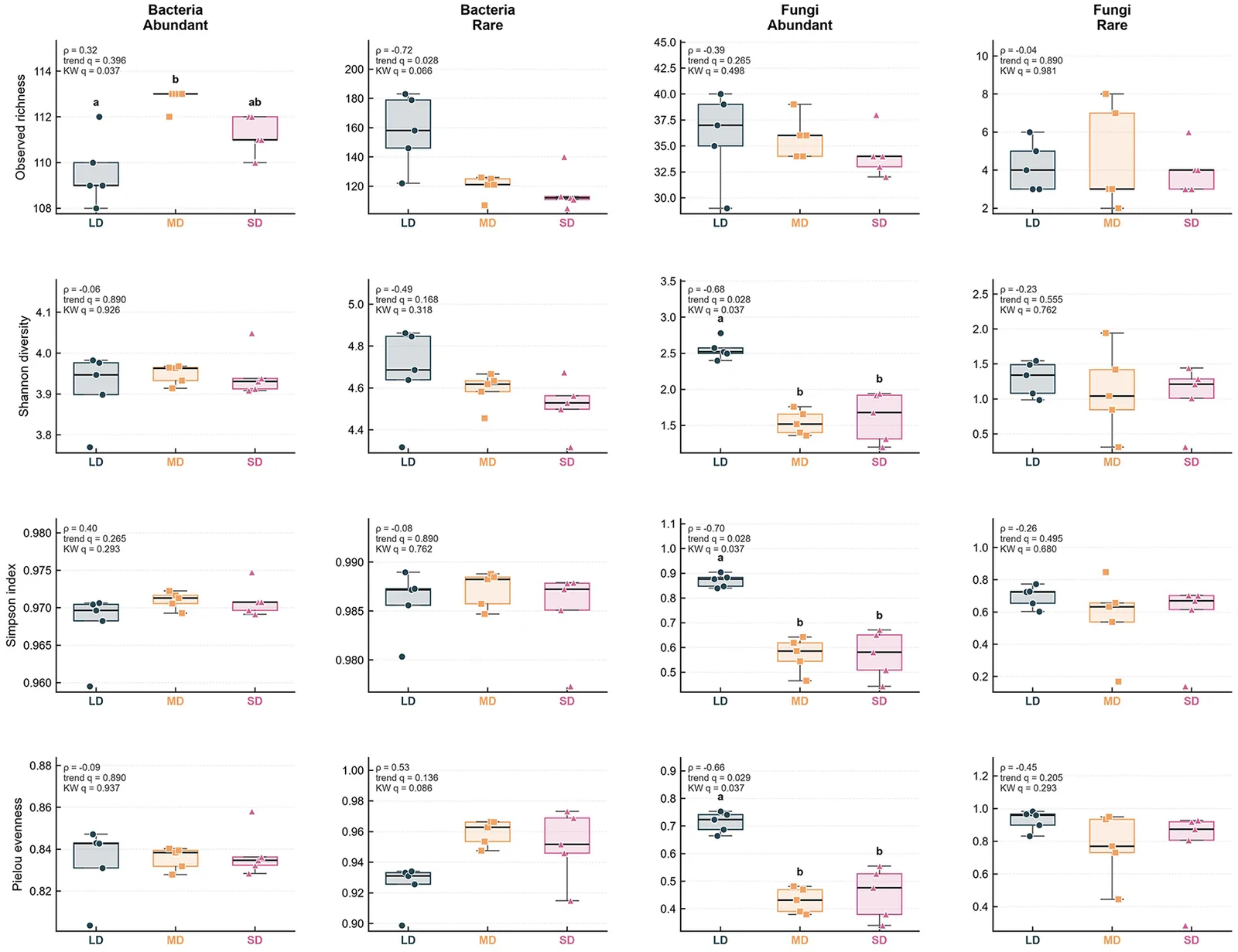
Alpha diversity of abundant and rare microbial subcommunities along the alpine marsh wetland degradation gradient. Observed richness, Shannon diversity, Simpson index, and Pielou's evenness are shown for abundant and rare bacterial and fungal subcommunities. Points represent individual samples (*n* = 5 per degradation level). Insets report Spearman's ρ, FDR-adjusted trend q, and Kruskal–Wallis *q* values.

In abundant fungi, Shannon diversity, Simpson diversity, and Pielou's evenness decreased along the gradient (trend *q* = 0.028–0.029; Kruskal–Wallis *q* = 0.037). Dunn tests separated LD from both MD and SD, while MD and SD did not differ. No rare-fungal alpha-diversity metric was significant after FDR correction. Thus, the supported patterns were a monotonic richness decline in rare bacteria and coordinated diversity and evenness declines in abundant fungi.

### 3.3 Compositional reorganization of abundant and rare subcommunities

PCoA revealed distinct patterns of compositional reorganization among abundant and rare subcommunities along the degradation gradient (Figure 4; Supplementary Table S3). The abundant bacterial subcommunity differed significantly among LD, MD, and SD (Figure 4a; PERMANOVA R^2^ = 0.730, *q* < 0.001), with all pairwise comparisons among the three degradation levels being significant. Rare bacteria also exhibited significant overall compositional reorganization (Figure 4b; R^2^ = 0.637, *q* < 0.001); however, the differences were mainly attributable to the separation of LD from MD and SD, whereas MD and SD did not differ significantly.

**Figure 4:**
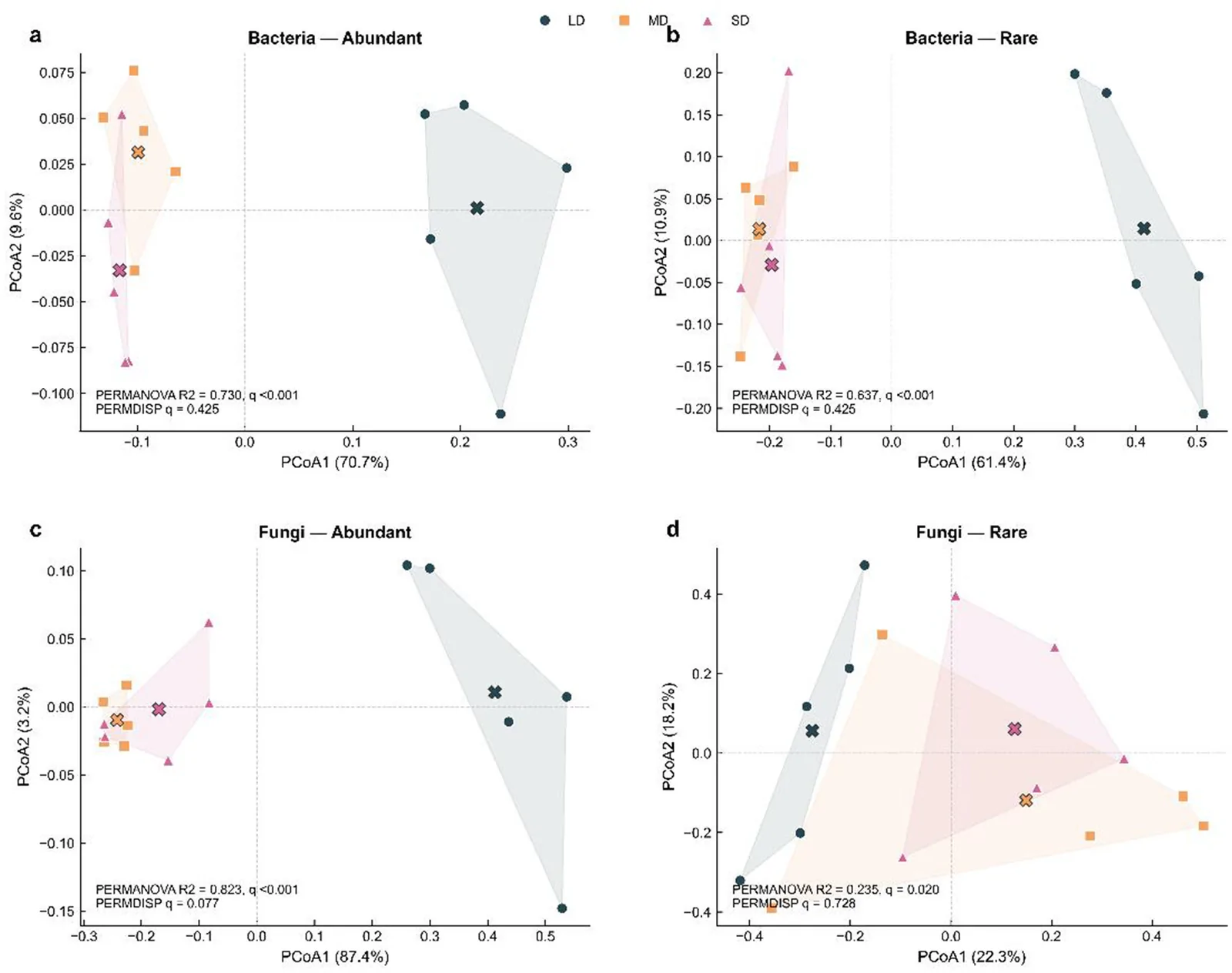
Compositional turnover of abundant and rare bacterial and fungal subcommunities. PCoA was performed using Bray–Curtis dissimilarities for abundant bacteria **(a)**, rare bacteria **(b)**, abundant fungi **(c)**, and rare fungi **(d)**. Shaded polygons represent group convex hulls and crosses indicate group centroids. PERMANOVA and PERMDISP statistics are shown in each panel.

Abundant fungi showed the strongest degradation-associated compositional differentiation (Figure 4c; R^2^ = 0.823, *q* < 0.001), with LD significantly separated from both MD and SD, whereas MD and SD were comparatively similar. The overall composition of rare fungi also differed significantly among degradation levels (Figure 4d), although the proportion of variation explained was substantially lower (R^2^ = 0.235, *q* = 0.020). PERMDISP was nonsignificant after FDR correction for all four subcommunities, indicating that the observed compositional differences primarily reflected shifts in group centroids rather than differences in within-group dispersion. Overall, abundant bacterial and fungal subcommunities exhibited the strongest compositional responses to degradation, whereas the response of rare fungi was comparatively weak.

### 3.4 β-diversity components and ecological niche breadth

Absolute incidence-based beta-diversity components differed strongly between abundance classes (Figure 5; Supplementary Table S5). Abundant bacterial total Jaccard dissimilarity was close to zero in LD, MD, and SD (0.022, 0.004, and 0.016); although nestedness-resultant dissimilarity formed the larger proportional component, its absolute magnitude was only 0.019, 0.004, and 0.012. This pattern indicates strong within-category compositional consistency rather than pronounced nestedness-driven restructuring. Rare bacterial total dissimilarity was higher and similar among LD, MD, and SD (0.282, 0.274, and 0.282). Turnover increased numerically from 0.075 in LD to 0.129 in MD and 0.111 in SD, while nestedness-resultant dissimilarity was 0.208, 0.145, and 0.171; these non-independent pairwise summaries were interpreted descriptively.

**Figure 5:**
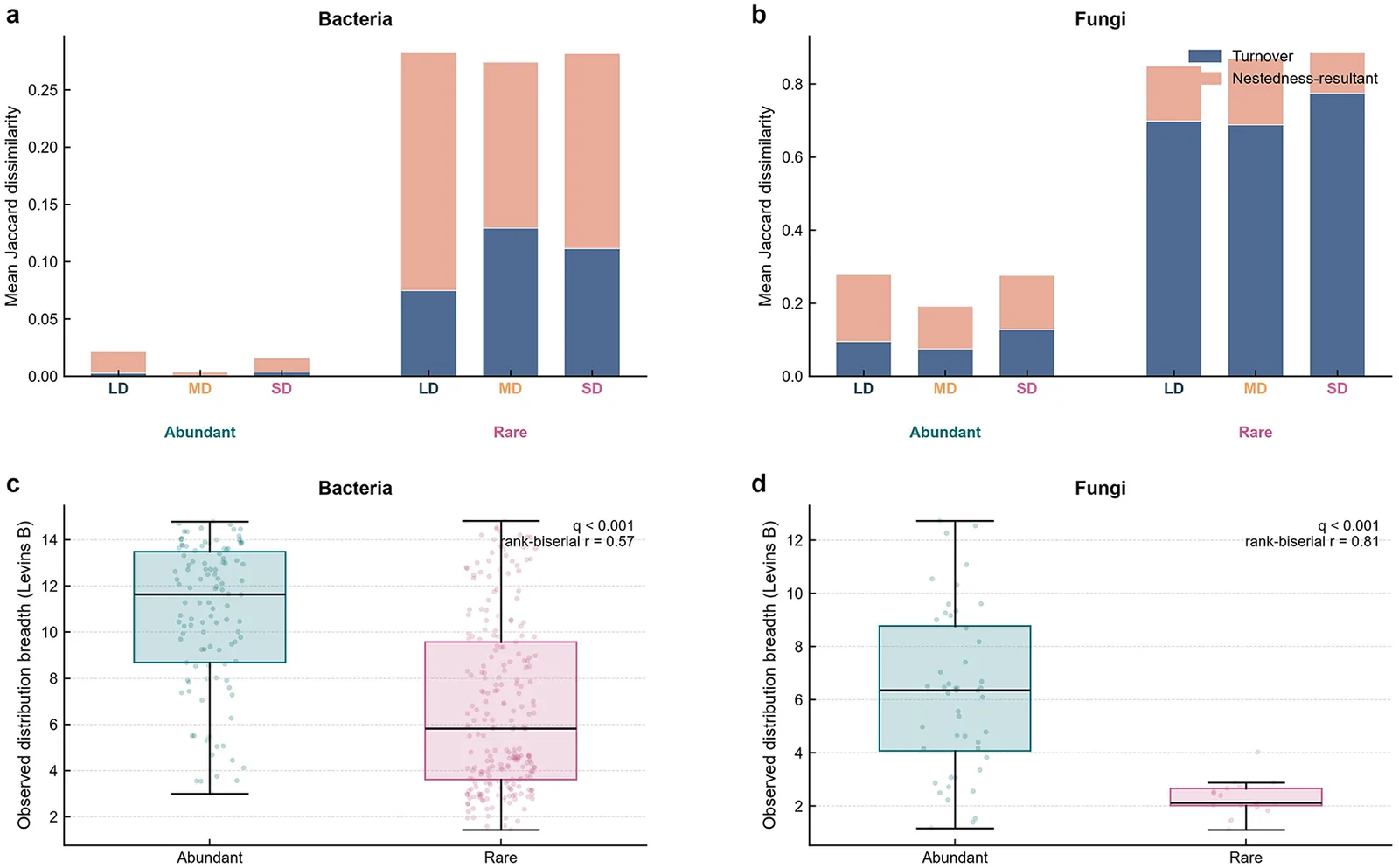
β-diversity components and niche breadth of abundant and rare microbial subcommunities. **(a)** Mean within-group Jaccard dissimilarity of bacterial subcommunities, partitioned into turnover and nestedness-related components. **(b)** Mean within-group Jaccard dissimilarity of fungal subcommunities, partitioned into turnover and nestedness-related components. **(c)** Levins' niche breadth of abundant and rare bacterial genera. Points represent individual genera; *q* values were obtained from FDR-adjusted Wilcoxon tests. **(d)** Levins' niche breadth of abundant and rare fungal genera.

Abundant fungal total Jaccard dissimilarity was 0.278, 0.192, and 0.276 in LD, MD, and SD, respectively. Rare fungi showed much higher sample-to-sample dissimilarity (0.849, 0.869, and 0.886), dominated by absolute turnover components of 0.698, 0.688, and 0.775. The data therefore support high rare-fungal compositional variability, but not a confirmatory monotonic trend. Abundant genera had greater Levins B than rare genera in both bacteria and fungi (both *q* < 0.001), corresponding to broader observed distributions across the 15 samples. Because detection probability declines with abundance, both distribution breadth and incidence-based beta diversity should be interpreted with this limitation in mind.

### 3.5 Indicator genera across degradation levels

IndVal analysis identified 70 degradation-level indicator genera that remained significant after FDR correction (Figure 6a). LD contained the largest number of indicator genera, particularly among rare bacteria, for which 37 LD-associated indicator genera were identified. In addition, LD contained 10 abundant bacterial and 10 abundant fungal indicator genera. Representative abundant bacterial indicators of LD included *Rubrivivax, Candidatus Deferrimicrobium, Hydrogenophaga, Thiobacillus, Anaerolinea*, and *Gallionella*, whereas abundant fungal indicators included *Cryphonectria, Diaporthe, Xylogone, Bimuria, Periconia*, and *Pyrenophora*.

**Figure 6:**
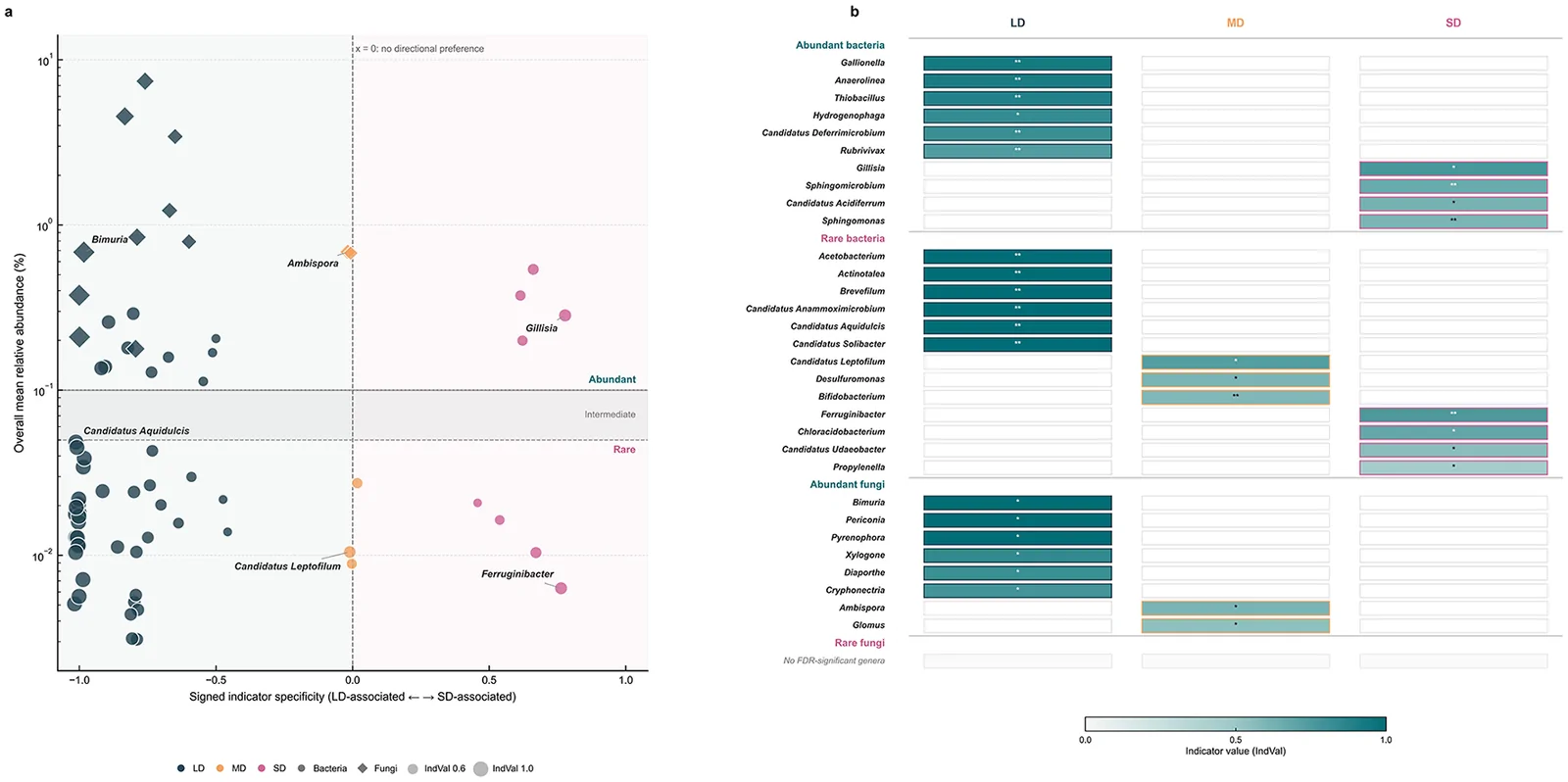
Indicator genera associated with alpine marsh wetland degradation levels. Significant bacterial **(a)** and fungal **(b)** indicator genera associated with LD, MD, or SD wetlands. Bubble size represents the Indicator Value, fill color denotes degradation level, and outline color distinguishes abundant and rare taxa. Only genera with FDR-adjusted *q* < 0.05 are shown.

MD contained comparatively few significant indicators (Figure 6b), including three rare bacterial genera and the abundant fungal genera *Glomus* and *Ambispora*. SD contained four abundant bacterial and four rare bacterial indicator genera, with representative abundant bacterial indicators including *Sphingomonas, Candidatus Acidiferrum, Sphingomicrobium*, and *Gillisia*. No FDR-significant fungal indicator genera were detected for SD, and no rare fungal indicator genera were identified. The pronounced concentration of indicator genera in LD, particularly within the rare bacterial subcommunity, indicates that degradation was accompanied not only by replacement among abundant taxa but also by declines or redistribution of numerous rare taxa associated with lightly degraded conditions. Indicator analysis identified distinct degradation-associated genera among microbial subcommunities (Supplementary Table S6).

### 3.6 Environmental explanation of abundant and rare subcommunity composition

PCA of soil physicochemical properties showed that PC1 and PC2 explained 53.5% and 25.1% of the total environmental variation, respectively, accounting for 78.6% in total (Figure 7). Samples from LD, MD, and SD were progressively separated along the major ordination axes in environmental space. LD samples were generally associated with higher soil moisture and greater availability of *N, P*, and *K*, whereas SD samples were positioned toward higher soil pH, with MD occupying an intermediate position. These patterns indicate the formation of a distinct composite soil environmental gradient corresponding to increasing degradation severity.

**Figure 7:**
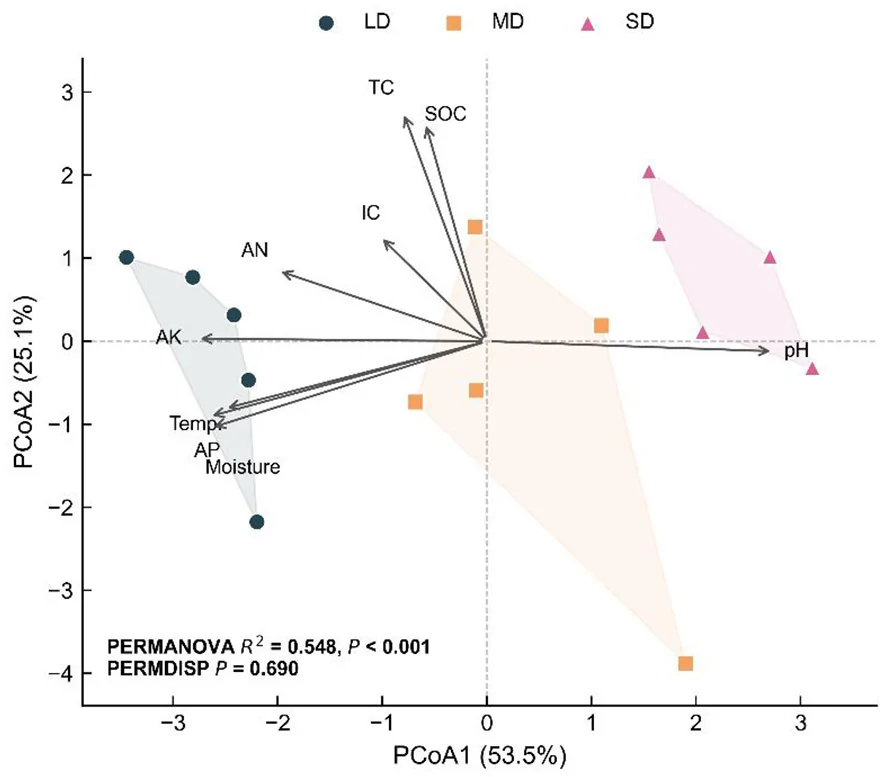
PCA of nine standardized soil physicochemical variables across LD, MD, and SD wetlands. Arrows indicate variable loadings.

Mantel analyses revealed that abundant and rare bacterial subcommunities (Figure 8), as well as abundant fungal subcommunities, were significantly associated with multiple soil properties, particularly pH, available phosphorus, available potassium, soil moisture, and soil temperature. In contrast, rare fungal communities showed no significant associations with the measured soil variables. dbRDA based on soil PC1 and PC2 further demonstrated that the environmental gradient significantly explained compositional variation in abundant bacteria, rare bacteria, and abundant fungi. The adjusted proportion of explained variation was 51.6% for abundant bacteria, 41.4% for rare bacteria, and 55.1% for abundant fungi, with all relationships remaining significant after FDR correction (all *q* < 0.001). In comparison, the adjusted explained variation for rare fungi was only 0.6% and was not significant (*q* = 0.438). Accordingly, the unexplained fraction was larger for rare bacteria than for abundant bacteria, indicating that rare bacterial composition was less predictable from the measured soil gradient.

**Figure 8:**
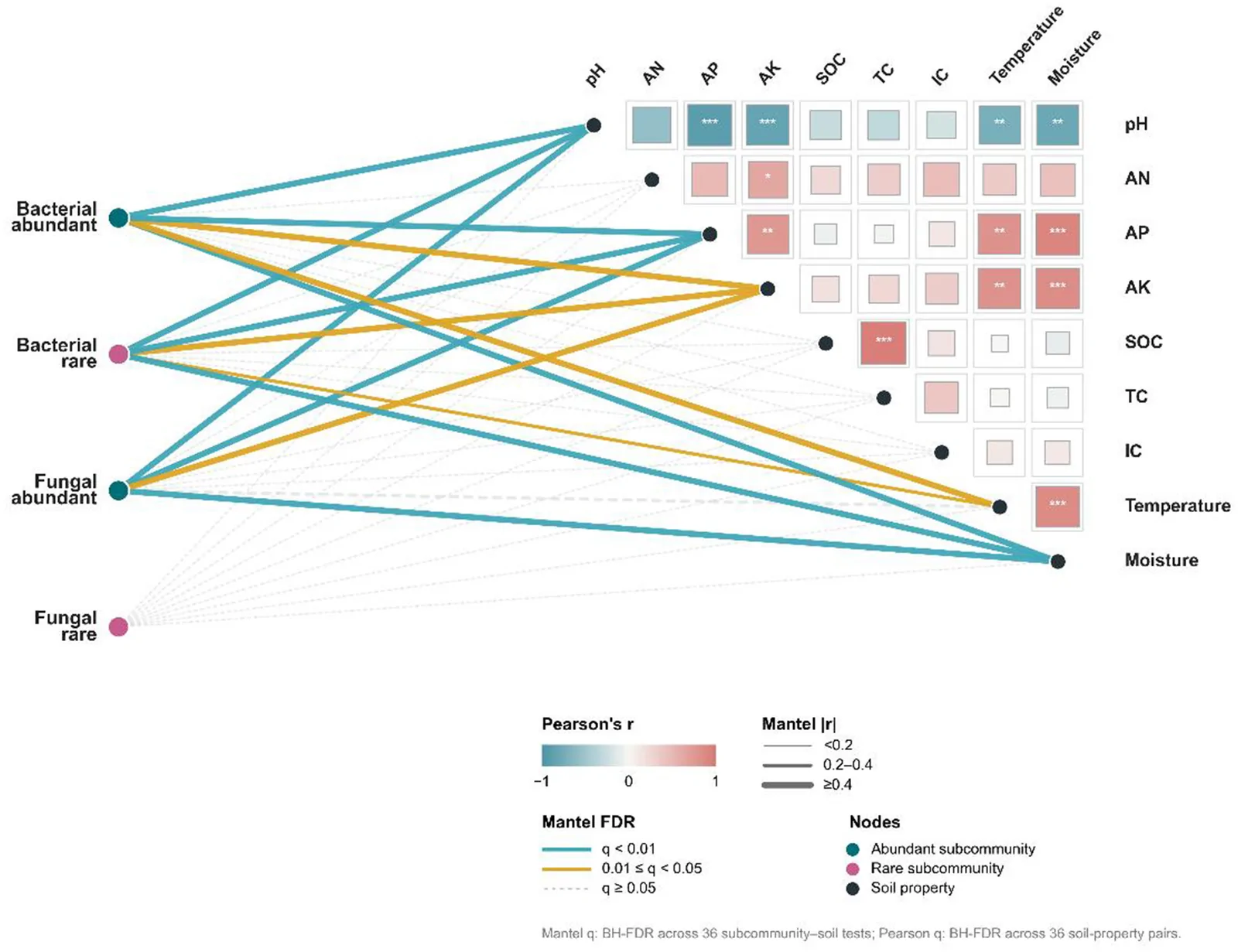
Associations between soil environmental gradients and microbial subcommunities. Variation in abundant and rare bacterial and fungal community composition explained by soil PC1 and PC2, based on dbRDA. Bars show adjusted R^2^ values, with FDR-adjusted permutation *q* values displayed alongside.

Together, these analyses showed that abundant bacterial and fungal subcommunities were more closely associated with the soil degradation gradient, whereas rare bacteria showed moderate environmental associations and rare fungi exhibited substantial compositional variation that could not be explained by the measured soil properties.

## 4 Discussion

### 4.1 Asymmetric responses of abundant and rare subcommunities

Microbial responses to ecosystem degradation are often evaluated at the whole-community level, yet our results demonstrate that degradation responses differed substantially between abundant and rare subcommunities. Although rare taxa represented a larger proportion of bacterial taxonomic diversity, abundant taxa accounted for the majority of total community abundance in both bacteria and fungi. This asymmetric distribution between taxonomic richness and relative abundance is consistent with the concept of the microbial rare biosphere, in which a large number of low-abundance taxa coexist with a limited number of dominant taxa that contribute disproportionately to community biomass and potentially to ecosystem processes (Lynch and Neufeld, 2015; Jousset et al., 2017). Such abundance–diversity decoupling has been widely observed across aquatic and terrestrial ecosystems, indicating that microbial communities are structured by both dominant ecological strategies and a diverse pool of low-abundance taxa that may serve as reservoirs of functional potential (Pedrós-Alió, 2012).

The contrasting α-diversity responses observed among abundant and rare subcommunities further indicate that degradation does not induce a uniform biodiversity decline. In our study, rare bacterial richness decreased significantly along the degradation gradient, whereas abundant fungal diversity and evenness declined, while abundant bacteria and rare fungi remained comparatively stable. Similar abundance-dependent responses have been reported in grasslands, soils, and aquatic systems, where rare and abundant microorganisms differed in their sensitivity to environmental gradients because of differences in habitat preference, dispersal limitation, and ecological specialization (Jiao et al., 2017). However, rare taxa should not be universally considered as more vulnerable components of microbial communities. Such ecological specialization does not necessarily imply reduced persistence, because taxa with narrow niches may persist through dormancy, spatial refugia, or temporal habitat variability.Some rare microorganisms can persist through dormancy, temporal fluctuations, or functional redundancy, allowing them to maintain long-term stability despite environmental disturbance (Lennon and Jones, 2011; Shade et al., 2014). Therefore, the loss of rare bacterial richness observed here likely reflects selective restructuring of specific rare taxa rather than a general collapse of the rare biosphere.

Differences in abundance-class responses may arise from contrasting ecological strategies. Abundant taxa generally occupy broader environmental niches and experience greater dispersal opportunities, which can facilitate their persistence across heterogeneous environments (Nemergut Diana et al., 2013; Wu et al., 2017). In contrast, rare taxa often exhibit narrower distributions and stronger habitat associations, making them potentially more responsive to environmental filtering but also more dependent on local microhabitat conditions (Pedrós-Alió, 2012; Jousset et al., 2017). The distinct responses of abundant and rare subcommunities observed in alpine marsh wetlands therefore suggest that degradation restructures microbial diversity through selective redistribution among abundance classes rather than through a simple reduction in total microbial diversity.

### 4.2 Community turnover and ecological specialization

The contrasting niche breadth and β-diversity patterns between abundant and rare microbial subcommunities indicate that degradation-associated restructuring involved distinct ecological strategies rather than uniform responses across microbial taxa. In our study, rare bacterial and fungal taxa exhibited narrower niche breadths than abundant taxa, while rare fungal communities showed particularly high within-group dissimilarity dominated by taxonomic turnover. These patterns suggest that rare taxa represent a more spatially variable component of alpine marsh wetland microbiomes, with distributions potentially associated with localized habitat conditions. Similar abundance-dependent differences in niche breadth and habitat specialization have been observed across microbial ecosystems, where abundant taxa often occupy broader ecological ranges, whereas rare taxa may exhibit stronger associations with specific environmental contexts (Barberán et al., 2014; Logares et al., 2020; Mohapatra et al., 2023). However, abundance categories should not be interpreted as fixed ecological strategies because the relationship between rarity, specialization, and environmental adaptability varies among ecosystems (Gubry-Rangin et al., 2024). The high turnover of rare fungi observed here therefore likely reflects enhanced compositional variability under heterogeneous degradation conditions rather than direct evidence that degradation alone drives rare taxa replacement. While soil environmental gradients explained substantial variation in abundant bacteria, rare bacteria, and abundant fungi, rare fungal composition showed limited relationships with the measured environmental variables. This pattern is consistent with previous findings that abundant microorganisms generally exhibit broader environmental adaptation and stronger environmental associations than their rare counterparts (Wan et al., 2021; Wang X. et al., 2023). Community turnover generally emerges from the combined influence of environmental filtering, dispersal processes, and ecological drift (Vellend, 2010; Stegen et al., 2013), and the relative contribution of these processes may differ among microbial groups and environmental gradients.

The weak association between rare fungal composition and measured soil variables further indicates that rare microbial dynamics may involve environmental dimensions not captured by bulk soil measurements. While soil environmental gradients explained substantial variation in abundant bacteria, rare bacteria, and abundant fungi, rare fungal composition showed limited relationships with measured environmental variables. This pattern suggests that rare fungal assemblages may respond to fine-scale habitat heterogeneity, plant-derived resources, or other unmeasured factors that are difficult to characterize using conventional soil properties. Similar observations have shown that rare microbial fractions may exhibit distinct assembly processes and ecological associations compared with abundant taxa (Cottenie, 2005; Chase and Myers, 2011; Jia et al., 2018). Furthermore, the concentration of indicator genera within rare bacterial communities, particularly in lightly degraded wetlands, suggests that rare taxa may provide sensitive ecological signals of habitat transitions. Nevertheless, indicator taxa should be interpreted as ecological associations rather than functional drivers of degradation. Together, these results indicate that abundant taxa may contribute to microbial community persistence through broader environmental distributions, whereas rare taxa contribute to community variability through localized occurrence and higher replacement rates, highlighting the importance of incorporating abundance-based ecological strategies when evaluating microbial responses to alpine marsh wetland degradation.

### 4.3 Distinct environmental filtering of bacteria and fungi

Environmental filtering along the degradation gradient differed among bacterial and fungal subcommunities, suggesting that abundant and rare taxa were not equally constrained by changing soil conditions. In our study, soil pH, moisture, available nutrients, and temperature collectively characterized the degradation-related environmental gradient, with abundant bacteria, rare bacteria, and abundant fungi showing stronger associations with these environmental changes than rare fungi. Soil pH is widely recognized as a major determinant of microbial community composition because it regulates nutrient availability, physiological tolerance, and competitive interactions among microorganisms, with bacterial communities often showing stronger pH responses than fungal communities (Rousk et al., 2010). Declining soil moisture and nutrient availability during wetland degradation may further alter microbial habitats by modifying substrate diffusion, oxygen conditions, and resource accessibility, thereby imposing selective pressures on taxa with different ecological strategies (Manzoni et al., 2012; Evans and Wallenstein, 2014). The stronger environmental associations observed for abundant taxa suggest that these groups may track broad degradation-related habitat changes, whereas the weaker responses of rare fungi indicate that their distributions may depend more strongly on localized microhabitats or unmeasured ecological factors.

The contrasting environmental responses between bacterial and fungal subcommunities may also reflect differences in resource acquisition strategies and ecological niches. Bacteria generally respond rapidly to changes in inorganic nutrients and soil physicochemical conditions because of their short generation times and dependence on readily available substrates, whereas fungi often occupy broader resource niches through saprotrophic and symbiotic strategies (Strickland and Rousk, 2010). In alpine marsh wetlands, changes in vegetation cover, soil moisture, and nutrient availability may therefore filter bacterial and fungal communities through different pathways rather than applying a uniform environmental constraint. Although soil carbon pools (SOC, TC, and IC) did not show significant changes across degradation levels in our study, carbon availability may still influence microbial organization through changes in substrate quality, plant inputs, and carbon accessibility that are not fully reflected by bulk carbon measurements. The absence of detectable changes in bulk carbon pools may reflect the slower turnover of soil carbon stocks relative to microbial community reorganization. Similar decoupling between microbial community shifts and bulk soil carbon changes has been reported during ecosystem transitions, where microbial responses can precede detectable changes in ecosystem carbon stocks (Kallenbach et al., 2016). Therefore, the stronger environmental coupling of abundant bacteria and fungi, compared with rare fungi, suggests that degradation-associated microbial restructuring involves differential environmental filtering among subcommunities rather than a single dominant environmental driver.

### 4.4 Ecological implications for degradation assessment and restoration monitoring

The contrasting ecological characteristics of abundant and rare microbial subcommunities provide implications for improving degradation assessment in alpine marsh wetlands. In our study, rare bacterial communities contained a large proportion of degradation-associated indicator genera, particularly under lightly degraded conditions, suggesting that rare taxa may capture subtle habitat transitions that are not fully reflected by dominant microbial groups. Microbial communities have increasingly been proposed as indicators of ecosystem condition because they integrate responses to environmental disturbance, resource availability, and ecological recovery processes (Harris, 2009; Hart et al., 2020). In particular, low-abundance taxa may contribute disproportionally to temporal and environmental changes in microbial diversity, acting as a potential reservoir of ecological responses under changing conditions (Shade et al., 2014; Pascoal et al., 2021). However, rare taxa should be interpreted as sensitive ecological indicators rather than direct drivers of degradation, because their distributions reflect the combined influence of habitat conditions, dispersal processes, and population dynamics. Therefore, incorporating rare microbial fractions together with environmental measurements may improve the sensitivity of degradation monitoring, especially in ecosystems where microbial restructuring occurs before detectable changes in ecosystem-level properties.

Assessing microbial recovery solely based on abundant taxa may underestimate the complexity of community reorganization during alpine marsh wetland restoration. Although abundant bacteria and fungi represented the major component of microbial abundance and showed stronger relationships with environmental gradients, rare taxa contributed substantially to community diversity and exhibited greater compositional turnover. This distinction indicates that abundant taxa may better represent dominant ecological functions, whereas rare taxa provide additional information on community flexibility and the maintenance of microbial diversity. Recent restoration studies have demonstrated that microbial community composition can be incorporated into restoration trajectory assessment and reference-state evaluation (Liddicoat et al., 2019, 2022). Meanwhile, microbial recovery does not necessarily involve a simple return of dominant taxa, but may require the reassembly of diverse microbial pools and ecological interactions (Pesce et al., 2017). Therefore, restoration evaluation of alpine marsh wetlands should consider both abundant and rare microbial pools to obtain a more complete understanding of microbial stability and recovery potential.

## 5 Conclusions

Alpine marsh wetland degradation reorganized soil microbial communities in a subcommunity-specific manner. Abundant taxa were more strongly associated with the soil environmental gradient, whereas rare taxa exhibited greater spatial turnover and a smaller proportion of compositional variation explained by the measured soil variables. The concentration of degradation-associated indicator genera within rare bacterial communities and the pronounced turnover of rare fungal communities further highlight that different rare microbial fractions provide distinct ecological signals of wetland degradation. These contrasting responses indicate that whole-community analyses alone may obscure important differences between abundant and rare taxa in their responses to degradation, ecological niche characteristics, and mechanisms of community maintenance.

## Data Availability

The data analyzed in this study are available in the NCBI (https://www.ncbi.nlm.nih.gov) under BioProject accession PRJNA1480021.
